# Single-cell RNA-sequencing analysis reveals MYH9 promotes renal cell carcinoma development and sunitinib resistance via AKT signaling pathway

**DOI:** 10.1038/s41420-022-00933-6

**Published:** 2022-03-22

**Authors:** Zhipeng Xu, Min Liu, Jin Wang, Kai Liu, Liuyu Xu, Demin Fan, Hui Zhang, Wenxin Hu, Dan Wei, Jianning Wang

**Affiliations:** 1 Department of Urology, Shandong Qianfoshan Hospital, Cheeloo college of Medicine, Shandong University, Jinan, Shandong China; 2 Department of Urology, The First Affiliated Hospital of Shandong First Medical University & Shandong Provincial Qianfoshan Hospital, Shandong medicine and Health Key Laboratory of Organ Transplantation and Nephrosis, Shandong Institute of Nephrology, Jinan, China; 3 Department of Urology, Changhai Hospital, Naval Medical University, Shanghai, China; 4 Department of Endocrinology and Metabology, The First Affiliated Hospital of Shandong First Medical University & Shandong Provincial Qianfoshan Hospital, Shandong Key Laboratory of Rheumatic Disease and Translational medicine, Shandong Institute of Nephrology, Jinan, China

**Keywords:** Renal cell carcinoma, Prognostic markers

## Abstract

Clear cell renal cell carcinoma (ccRCC) is a serious threat to human health worldwide, while its heterogeneity limits therapeutic success and leads to poor survival outcomes. Single-cell RNA-sequencing (scRNA-seq) is an important technology, which provides deep insights into the genetic characteristics of carcinoma. In this study, we profiled the gene expression of single cells from human ccRCC tissues and adjacent normal tissues using the scRNA-seq. We found that MYH9 was commonly upregulated in the ccRCC cell subgroup. Additionally, MYH9 was of highly expression in ccRCC tissues and predicted poor prognosis of ccRCC patients. MYH9 knockdown in ccRCC cells dampened their proliferative and metastatic potentials, whereas MYH9 overexpression enhanced these properties. In vivo, MYH9 also promoted ccRCC growth. Mechanistic studies showed that MYH9 played these vital roles through AKT signaling pathway. Furthermore, MYH9/AKT axis determined the responses of ccRCC cells to sunitinib treatment and might serve as a biomarker for sunitinib benefits in ccRCC patients. Thus, MYH9 might be a novel therapeutic target and prognostic predictor for ccRCC.

## Introduction

Renal cell carcinoma (RCC) is one of the top ten harmful tumors, and accounts for 2% to 3% of adult malignant tumors [1]. Clear cell RCC (ccRCC), the most common form of RCC, accounts for more than 90% of cases. Although most of patients with ccRCC have localized tumors, approximately 17% of patients present with metastatic ccRCC at the time of diagnosis [2]. Furthermore, up to 20% localized ccRCC patients develop metastasis or recurrence after surgical resection [3]. Although therapies of ccRCC have evolved considerably over the past decade, the prognosis is still poor, especially for ccRCC patients with metastasis [4]. Moreover, targeting drugs, such as sunitinib, have been used in patients with ccRCC. Unfortunately, the heterogeneity of ccRCC causes drugs resistance and serious consequences of targeted therapies [5]. Since treatment methods for metastasis and recurrence are limited and ineffective, there is an urgent need to gain insight into the molecular mechanisms of ccRCC proliferation and metastasis to support the development of more efficient therapeutic measures in the near future.

Single-cell RNA-sequencing (scRNA-seq) is a new technique for high-throughput sequencing and analysis of RNA at a single cell level and has significantly advanced our knowledge of cell biology [6]. In recent years, scRNA-seq was used to deciphering intra-tumoral heterogeneity across cell subpopulations such as ccRCC. Young et al. adopted scRNA-seq to identify specific normal cell correlated of renal cancer cell, revealing the precise cellular identities and compositions of human kindey tumors [7]. Zhang et al. constructed metastasis-associated genes by scRNA-seq and validated the model in a large number of ccRCC samples, which was of great value to predict the prognosis of patients with ccRCC and provide potential targets against metastatic ccRCC [8]. In the recent study, we identified 5 cell subgroups from renal cancer tissues and adjacent normal tissues using scRNA-seq clustering analysis. In order to identify the cancer promoter gene, we compared the differences between renal tube cells and renal cancer cells and found 5 differentially expressed genes (DEGs) that were significantly higher in renal cancer cells than that in renal tube cells. Among them, Myosin heavy chain 9 (MYH9) was the most remarkable DEG, closely related to ccRCC prognosis.

MYH9 is a skeleton protein that mediates actin based contractile motion [9]. As a skeleton protein, MYH9 has been reported to play a vital role in cell adhesion, polarity, and motility [10]. In recent years, a growing body of researches has reported the deregulation of MYH9 in cancers [11]. Previous studies have shown that overexpression of MYH9 lead to invasion and metastasis of gastric cancer cells [12]. In colorectal carcinoma tissues, the expression of MYH9 is significantly higher than that of adjacent tissues [13]. Another study showed overexpression of MYH9 could cause the recurrence of pancreatic cancer by downregulation of many tumor suppressor genes [14]. Conversely, MYH9 has been reported to act as a tumor suppressor in skin cancer and in head and neck squamous cell cancers [15]. However, to the best of our knowledge, there has been no research about the role of MYH9 in renal cell carcinoma.

In this study, we identified MYH9 as a key tumor promotor in ccRCC by scRNA-seq analysis and bioinformatics analysis. MYH9 high expression was found in ccRCC tissues and predicated for poor prognosis of ccRCC patients. Subsequent studies showed that MYH9 accelerated the proliferation and migration and promoted sunitib resistance of ccRCC. Further mechanistic study revealed that MYH9 could activate AKT signaling, further promoting ccRCC development and sunitib resistance. Summarily, this study is a first attempt to explain the potential tumorigenic effect of MYH9 on ccRCC.

## Results

### scRNA-seq analysis screening of DEGs and validation of DEGs

**Fig. 1:**
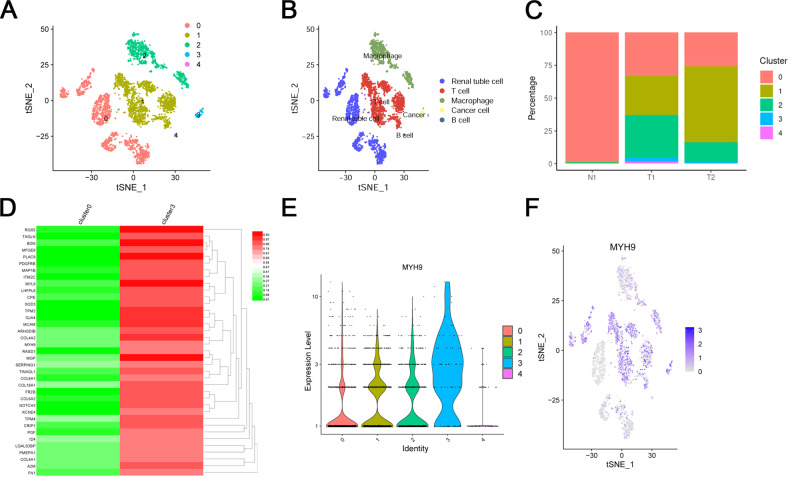
ScRNA-seq characterization and DEGs screening of ccRCC tissues. **A, B** Cluster map showing the assigned identity for each cluster defined. **C** Percentage of each cell cluster in each sample. **D** Heatmap showing the top 41 DEGs between renal tube cell (cluster 0) and cancer cell (cluster 3), obtained from the scRNA-seq analysis. **E, F** scRNA-seq analysis showing the expression level of MYH9 in 5 clusters, MYH9 is highly expressed in ccRCC cells (cluster 1).

A total of 3 ccRCC patients’ tissues, including 2 tumor tissues and 1 adjacent tissues, were used for scRNA-seq analysis. The quality control chart (Supplementary Fig. S1a) illustrated the range of the number of genes detected and the sequencing count for each cell. Accordingly, we selected cells with a percentage of mitochondrial sequencing count <5% for further analysis. The number of genes in the cells was positively correlated with the sum of gene expression of 0.85 (Supplementary Fig. S1b). Analysis of variance revealed the top 10 significantly DEGs in the cell sample, including SST, JCHAIN, IGFBP3, RGS5, S100A9, MZB1, ENPP2, TAGLN, S100A8 and IGKC (Supplementary Fig. S1c). Furthermore, we used the principal component analysis (PCA) method and screened the significantly correlated genes in each component. We mapped the cells into two dimensions based on the PC_1 to PC_20 components, and the five correct independent cell subpopulations indicated the preferable clustering efficiency during the PCA procedure (Supplementary Fig. S1d and S1e). Supplementary Figure S1f displayed the top 6 PC used to define cell subpopulations in the form of a heat map. Supplementary Figure S1g, Fig. 1a, and Fig. 1b further use the t-SNE algorithm to precisely cluster the populations of cells, successfully classifying the samples into five independent cell subgroups: cluster 0 (renal tuble cell), cluster 1 (T cell), cluster 2 (macrophage), cluster 3 (cancer cell), and cluster 4 (B cell). Moreover, in Fig. 1c, the percentage of cell clusters in each sample was shown. In order to identify the cancer promoter gene, we selected clusters 0 and 3 for further analysis. Compared to renal tube cell, the differential analysis revealed 41 DEGs was higher expressed in renal cancer cells with | log fold change (FC) | >1 and adj Pval < 0.05 (Fig. 1d and Supplementary Table S1). Before further analysis, we verified the accuracy of the 41 DEGs in the TCGA, Oncomine database, ualcan database, and GEPIA database. Finally, 5 genes that met our expectations were screened, including MYH9, A2M, FN1, COL6A1, and PGF (Fig. 1e, f and S2a–c). Subsequently, we measured the relative mRNA expression of the 5 genes in ccRCC cells (786-O, A498 ACHN, and Caki-1) and normal kidney cells (HK2 and 293 T), as shown in Supplementary Figure S3. We found that the mRNA expression of MYH9 in ccRCC cells was significantly higher than that in normal kidney cells. So we chose MYH9 for subsequent experiments to explore its impact on ccRCC progression.

### MYH9 is upregulated in ccRCC and correlates with patient prognosis

**Fig. 2:**
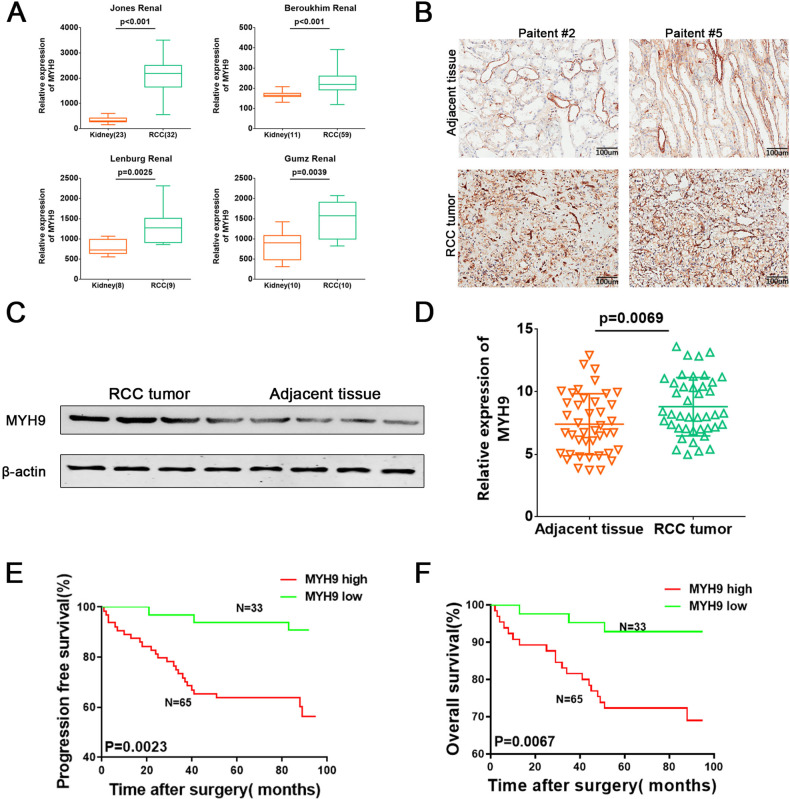
MYH9 is upregulated in ccRCC and correlates with patient prognosis. **A** MYH9 transcription in RCC (Oncomine). Levels of MYH9 mRNA were significantly higher in RCC than in adjacent kidney tissue. Shown are fold change, associated p values based on Oncomine 4.5 analysis. Box plot showing MYH9 mRNA levels in RCC and adjacent kidney tissue, respectively, the Jones Renal, Beroukhim Renal, Lenburg Renal, Gumz Renal. **B** IHC analysis of MYH9 in ccRCC tissues and adjacent normal tissues. Scale bar = 100 μm. **C** Western blotting analysis of MYH9 in ccRCC tissues and adjacent normal tissues. **D** RT-PCR analysis of MYH9 mRNA expression in human ccRCC tissues and adjacent normal tissues from 42 ccRCC patients. **E** Patients in comparative MYH9 high group (*n* = 65) had lower PFS than those in comparative MYH9 low group (*n* = 33). **F** Patients in comparative MYH9 high group (*n* = 65) had lower OS than those in comparative MYH9 low group (*n* = 33).

Data in the Oncomine database showed that the mRNA expression of MYH9 was significantly higher in RCC tissues than in normal kidney tissues (Fig. 2a). In our cohort 1, immunohistochemistry (IHC) staining and western blotting were performed and found that MYH9 was significantly upregulated in ccRCC tissues, compared to normal kidney tissues (Fig. 2b, c, Supplementary Table S2). The RT-PCR also showed that RCC tumor was of higher level of MYH9 than adjacent tissue in cohort 2 (Fig. 2d, Supplementary Table S3-4). Kaplan-Meier survival analysis (https://kmplot.com/analysis/) showed that high MYH9 expression was associated with poor overall survival and recurrence-free survival of RCC patients from GEO detabase (Fig. S4). Moreover, from our cohorts 2, we also found patients’ overall survival (OS) and progression-free survival (PFS) were significantly poorer in ccRCC with high MYH9 levels (Fig. 2e, f, Supplementary Table S5).

### MYH9 promotes the proliferation and migration of ccRCC cells

**Fig. 3:**
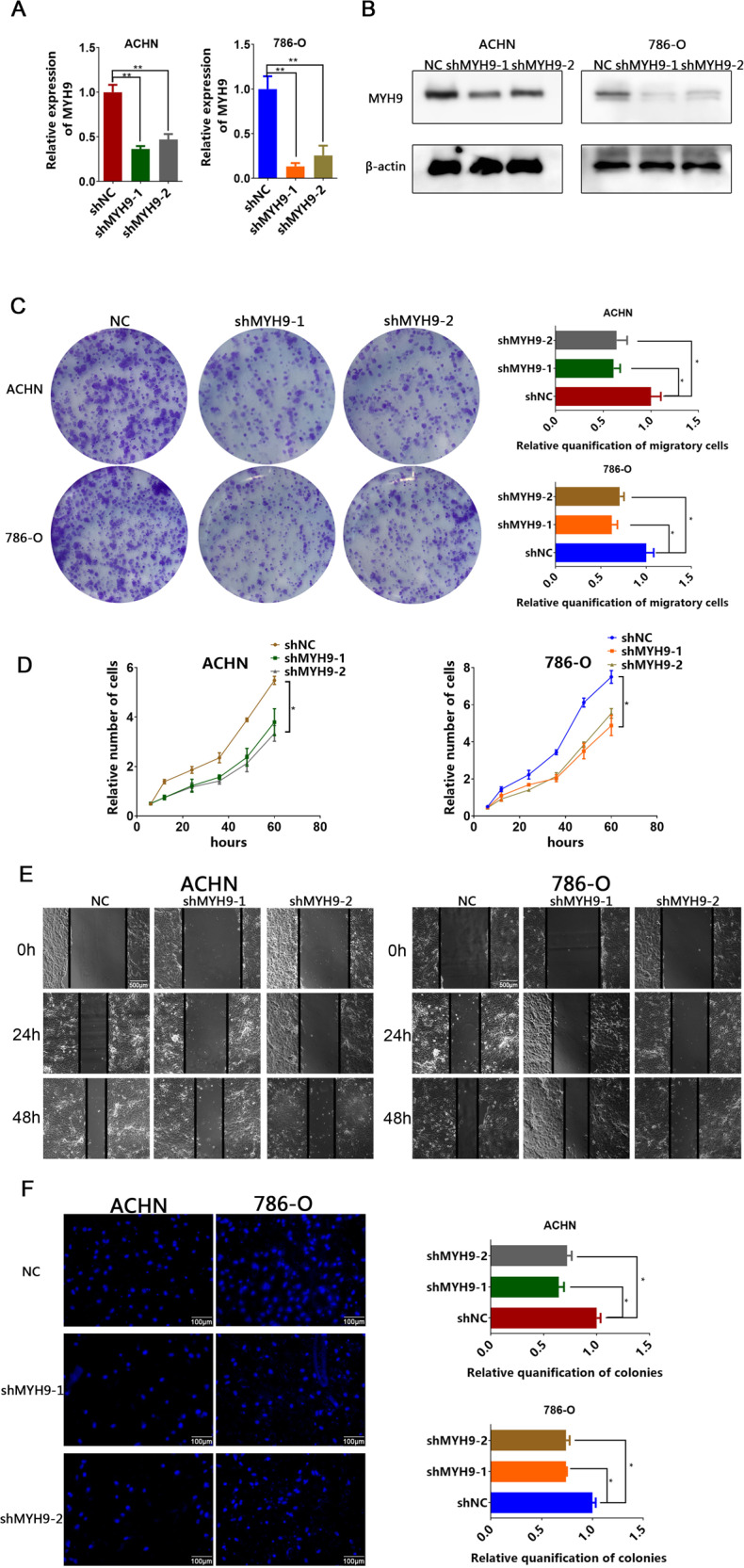
Downregulated MYH9 suppresses the potential for proliferation and migration of ccRCC cells. **A** RT-PCR analysis of MYH9 in ACHN (left) and 786-O (right) cells transfected with shMYH9-1, shMYH9-2 or shNC (*n* = 3). **B** Western blotting analysis of MYH9 in ACHN (left) and 786-O (right) cells transfected with shMYH9-1, shMYH9-2 or shNC (*n* = 3). Scale bar = 40 μm. **C** Plate colony formation assay of MYH9 knockdown and control ACHN and 786-O cells in 6-well plates for 10 days (*n* = 3). Average number of colonies and representative images were shown. **D** CCK8 assay of MYH9 knockdown and control ccRCC cells at indicated times. **E** Wound-healing assay of MYH9 knockdown and control ccRCC cells photographed at 0, 24 and 48 h after scratching. Scale bar = 500 μm. **F** Transwell migration of MYH9 knockdown and control ccRCC cells (*n* = 3). Scale bar = 100 µm. Average migrated number and representative images were shown.

**Fig. 4:**
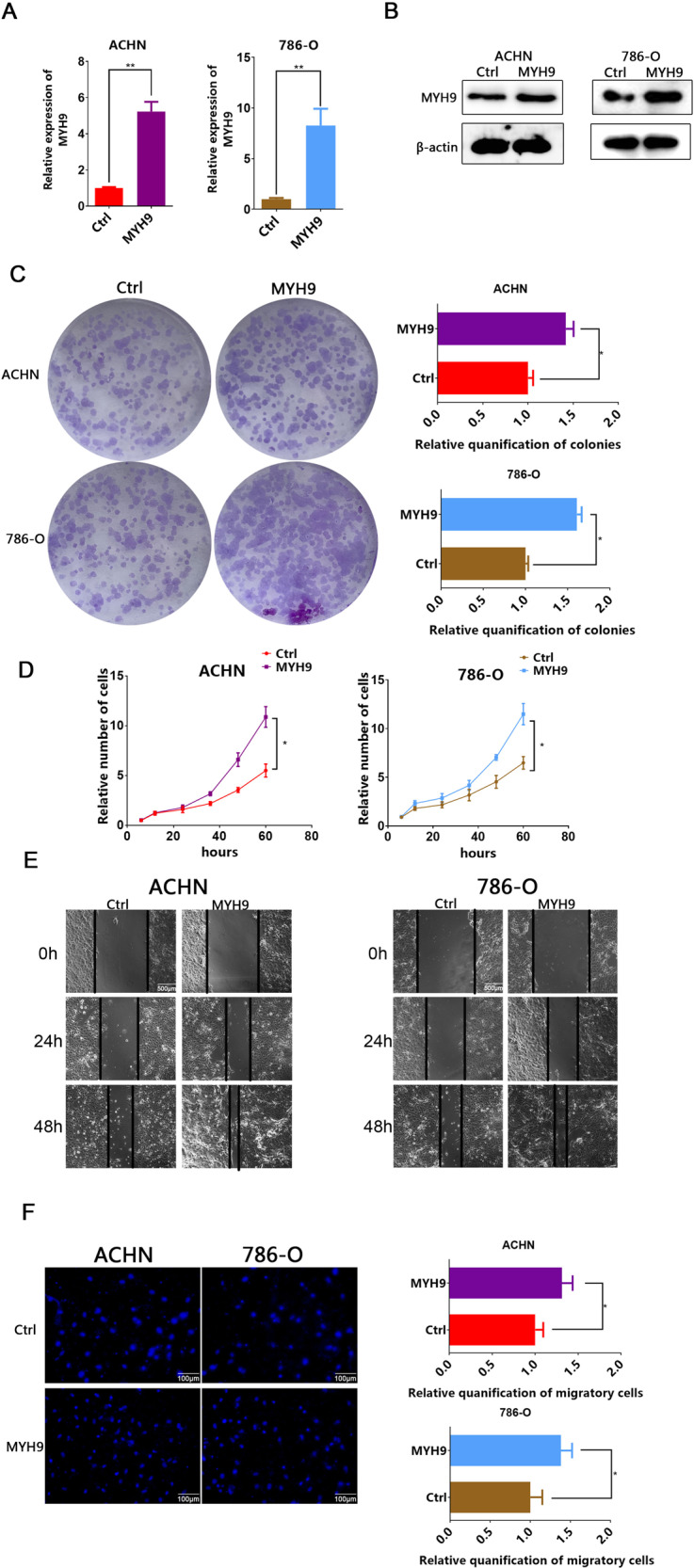
Upregulated MYH9 promotes the proliferation and migration of ccRCC cells. **A** RT-PCR analysis of MYH9 in ACHN (left) and 786-O (right) cells transfected with LV-MYH9 or LV-ctrl (*n* = 3). **B** Western blotting analysis of MYH9 in ACHN (left) and 786-O (right) cells transfected with LV-MYH9 or LV-ctrl (*n* = 3). Scale bar = 40 μm. **C** Plate colony formation assay of MYH9 overexpression and control ACHN and 786-O cells in 6-well plates for 10 days (*n* = 3). Average number of colonies and representative images were shown. **D** CCK8 assay of MYH9 overexpression and control ccRCC cells at indicated times. **E** Wound-healing assay of MYH9 overexpression and control ccRCC cells photographed at 0, 24 and 48 h after scratching. Scale bar = 500 μm. **F** Transwell migration MYH9 overexpression and control ccRCC cells (*n* = 3). Scale bar = 100 µm. Average migrated number and representative images were shown.

**Fig. 5:**
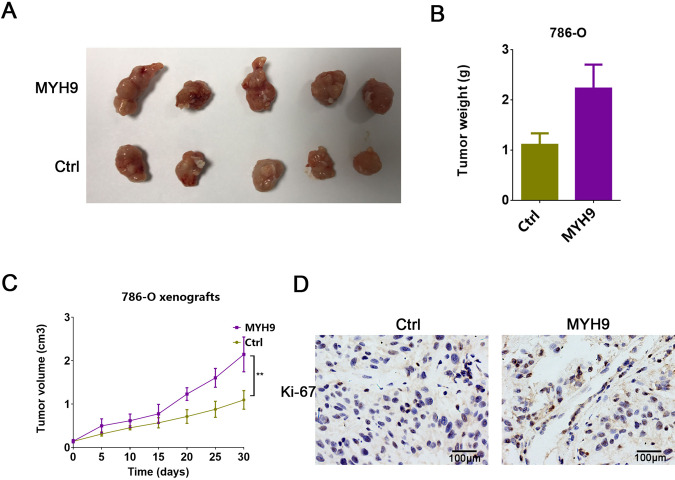
MYH9 enhances tumor growth in vivo. **A** Representative nude mice showing the morphology of the tumors derived from MYH9 overexpression and control 786-O cells. **B** Average weight of LV-MYH9 versus the control 786-O tumors. **C** The growth curve of LV-MYH9 versus the control 786-O tumors. **D** Ki-67 staining of LV-MYH9 versus the control 786-O tumors. Scale bar = 100 μm.

In attempt to explore the role of MYH9 in growth and metastasis of RCC cells, we silenced the expression of MYH9 with shRNA and overexpressed MYH9 with lentivirus-MYH9 (LV-MYH9) in two ccRCC cell lines (Figs. 3a, b and 4a, b). Cell growth was assessed in plate colony formation assay and cell counting kit-8 (CCK8) assay and we found MYH9 knockdown significantly inhibited the proliferation (Fig. 3c, 3d), while MYH9 overexpression promoted the proliferation of ccRCC cells (Figs. 3d, 4c). Furthermore, wound-healing assay and transwell migration assay revealed that knockdown of MYH9 significantly suppressed cell migration compared with that of control cells (Fig. 3e, f). In contrast, overexpression of MYH9 enhanced the migration of ccRCC cells (Fig. 4e, f). Subsequently, tumor proliferation was detected by subcutaneous injection into nude mice with ccRCC cell line in vivo. As showed in Fig. 5a, b, nude mice injected with MYH9 overexpression cells presented with higher tumor volumes and weights than those in control group (Fig. 5c). IHC staining showed that proliferation marker Ki-67 was of stronger level in overexpressed MYH9 tumors (Fig. 5d). Thus, MYH9 promoted the proliferation and migration of ccRCC cells in vitro and in vivo.

### MYH9 promotes ccRCC progression via activating AKT signaling pathway

**Fig. 6:**
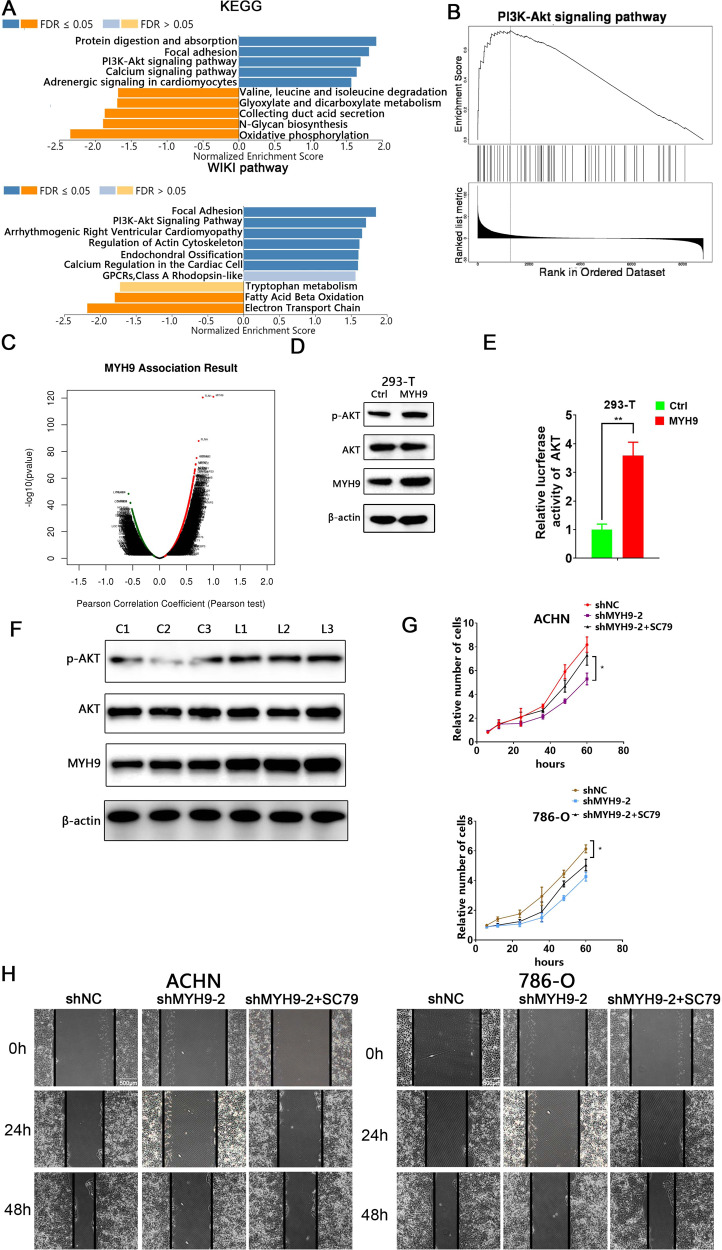
MYH9 promotes ccRCC progression via activating AKT signaling pathway. **A** A significantly enriched KEGG pathways and WIKI pathways target of MYH9 co-expression genes in RCC. **B** GSEA analysis showed impact of MYH9 on the AKT signaling pathway. **C** A Pearson test was used to analyze correlations between MYH9 and genes differentially expressed in RCC. **D** Western blotting analysis of the indicated proteins in 293-T cells transfected with LV-ctrl or LV-MYH9 (*n* = 3). **E** Luciferase activity assay of AKT luciferase reporter plasmids in 293-T cells transfected with LV-ctrl or LV-MYH9. **F** Western blotting analysis of the indicated proteins in 786-O xenografts transfected with LV-ctrl (C1-3) or LV-MYH9 (L1-3). **G** CCK8 assay of control and MYH9 knockdown ccRCC cells at indicated times along with vehicle or SC79 (5 μM) treatment. **H** Wound-healing assay of control and MYH9 knockdown ccRCC cells along with vehicle or SC79 (5 μM) photographed at 0, 24, and 48 h after scratching. Scale bar = 500 μm.

With the purpose to detect the underlying mechanism of MYH9 in ccRCC, KEGG and WIKI pathway analysis were performed. The results showed that the top three significant pathways in KEGG regarding the potential target of MYH9 were protein digestion and absorption, focal adhesion, PI3K-AKT signaling and top two significant gene annotations in WIKI pathway were focal adhesion and PI3K-AKT, respectively (Fig. 6a). In addition, GSEA analysis suggested a widespread impact of MYH9 on the AKT signaling pathway (Fig. 6b). We also analyzed mRNA sequencing data from 533 RCC patients in the TCGA by function module of LinkedOmics. The results showed that MYH9 expression exhibited a strong positive association with AKT1 (Pearson correlation = 0.44, *p* = 7.42e–27) expression levels in RCC tissues (Figs. 6c, s5 and s6). In our study, western blotting showed that phosphorylation of AKT was significantly upregulated in LV-MYH9 293-T cells (Fig. 6d). AKT reporter assay further confirmed that MYH9 up-regulation incurred higher level of AKT activation (Fig. 6e). Moreover, AKT was also remarkably activated in LV-MYH9 xenografts by western blotting (Fig. 6f). Therefore, we wondered whether MYH9 exerted these effects on ccRCC via AKT signaling pathway. As expected, the capacity of proliferation and migration were suppressed in shMYH9-2 ccRCC cells, while these abilities could be reversed by co-incubation with SC79, an AKT activator (Fig. 6g, h). Taken together, these results indicated that MYH9 promoted ccRCC progression through activating AKT signaling pathway.

### MYH9 promotes ccRCC sunitinib resistance via AKT signaling pathway in vitro

**Fig. 7:**
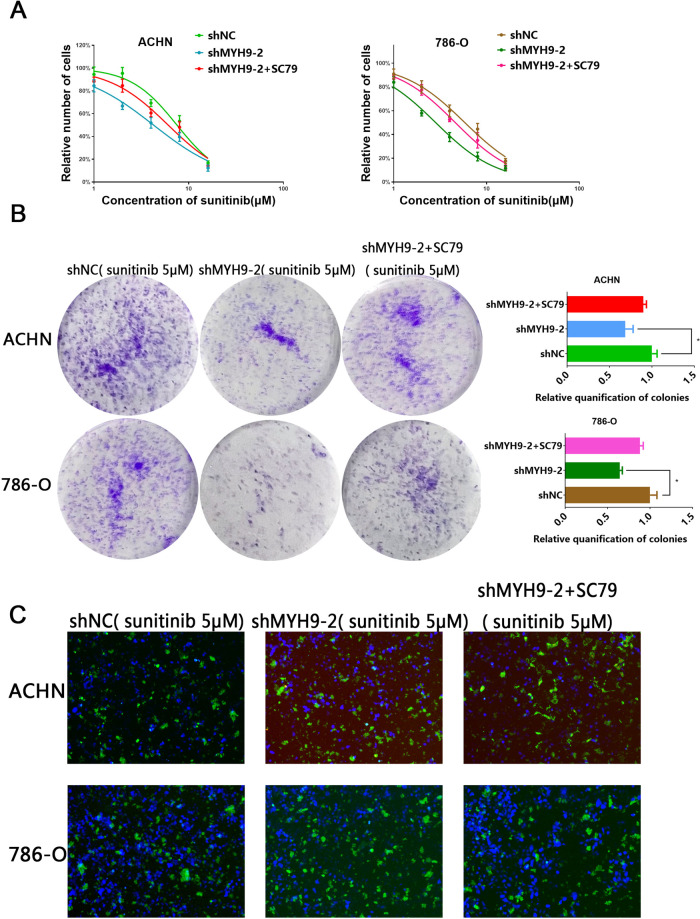
MYH9 promotes ccRCC sunitinib resistance via AKT signaling pathway in vitro. **A** CCK8 assay of control and MYH9 knockdown ccRCC cells upon sunitinib treatment along with vehicle or SC79 (5 μM) treatment for 48 h. **B** Plate colony formation assay of MYH9 knockdown ccRCC cells upon sunitinib treatment (5 μM) along with vehicle or SC79 (5 μM) treatment for 10 days (*n* = 3). **C** Apoptisis cells assay of control and MYH9 knockdown ccRCC cells upon sunitinib (5 μM) treatment in 6-well plates along with vehicle or SC79 (5 μM) treatment for 48 h (*n* = 3).

Growing evidence demonstrated that drug-induced activation of alternative RTKs may be regarded as a key contributor to the therapeutic resistance [16]. Now that AKT was the main downstream pathway of MYH9, we wondered whether MYH9 also took part in the resistance of sunitinib in ccRCC. CCK8 and colony formation assay revealed that MYH9 knockdown ccRCC cells presented an increased sensitivity to sunitinib treatment compared with control group, while activation of AKT remarkably restored sunitinib resistance (Fig. 7a, b). We also found that MYH9 knockdown led to the sensitivity of ccRCC cells to sunitinib by apoptosis cells assay and SC79 could significantly restored the resistance of ccRCC cells to sunitinib (Fig. 7c). Collectively, these data indicated that the MYH9-mediated AKT activation might be required for sunitinib resistance in ccRCC cells.

### High levels of MYH9 predicts poor responses to sunitinib in ccRCC patients

**Fig. 8:**
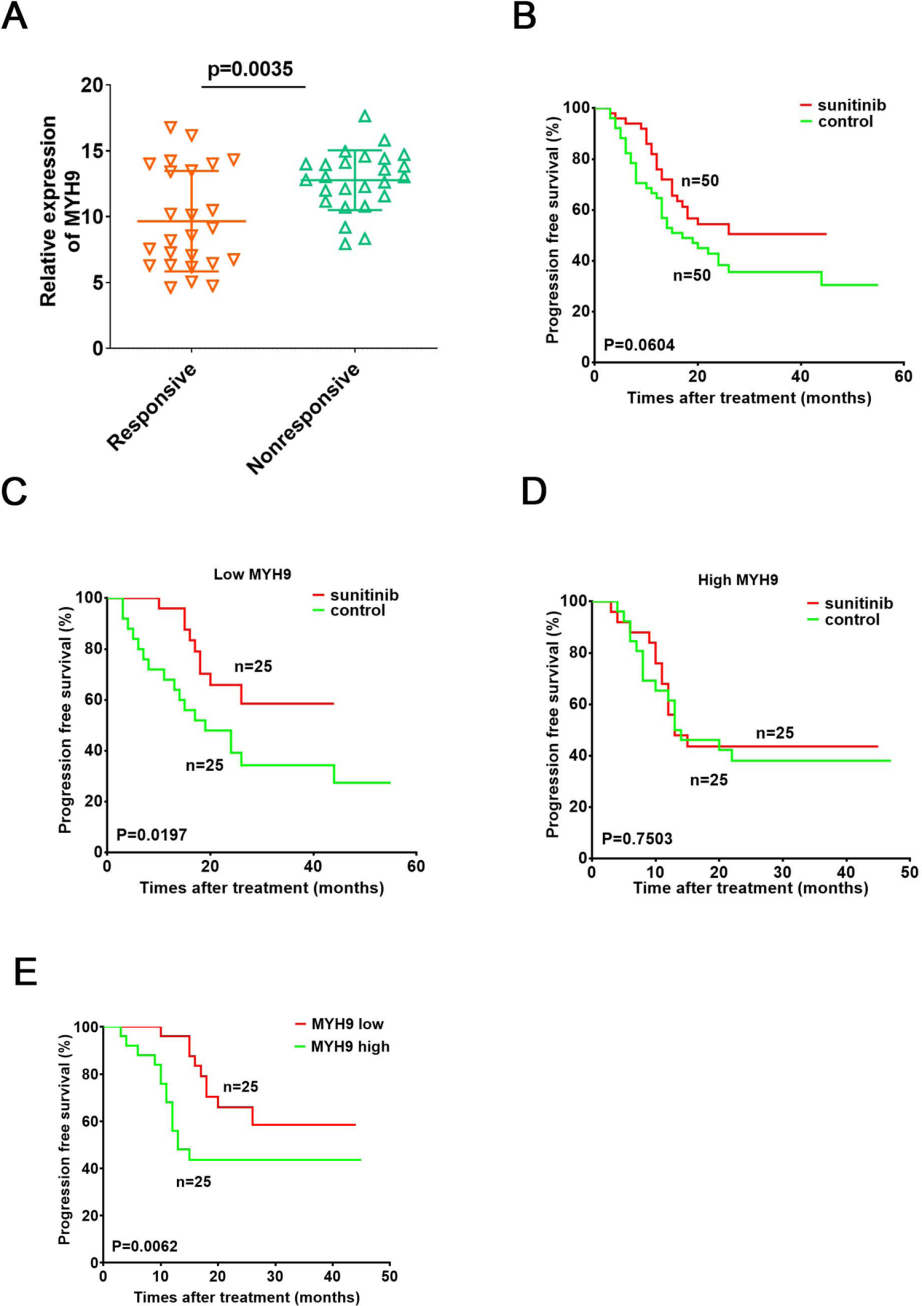
High levels of MYH9 predicts poor response to sunitinib in ccRCC patients. **A** RT-PCR analysis of MYH9 in tumor samples from an independent cohort of ccRCC patients with good (*n* = 25) or poor (*n* = 25) responses to sunitinib therapy. **B, C, D** Kaplan-Meier analysis of PFS in ccRCC patients with and without sunitinib therapy. **B** All patients regardless of MYH9 expression, **C** Patients with lower levels of MYH9 expression, **D** Patients with higher levels of MYH9 expression. **E** Kaplan-Meier analysis of PFS in ccRCC patients with low MYH9 expression and high MYH9 expression after sunitinb treatment. The median MYH9 expression level was used as the cutoff.

As MYH9 is functionally associated with sunitinib tolerance in ccRCC cells, we further explored whether MYH9 expression in tumor tissues was involved in the response of patients to sunitinib therapy. We detected the mRNA levels of MYH9 in 100 ccRCC samples from 50 sunitinib-treated patients and 50 no drug treated patients after surgery (Supplementary Table S6-7). In 50 sunitinib-treated ccRCC patients, the MYH9 levels were higher in the ccRCC tissues from poor response group than that from well response group (Fig. 8a). Sunitinib therapy provided limited benefits for the PFS of ccRCC patients (Fig. 8b). Intriguingly, patients with low MYH9 expression group displayed more notable improvement in PFS after receiving sunitinib than control group (Fig. 8c). While, compared with control group, high MYH9 expression patients did not present obvious clinical effect (Fig. 8d). Furthermore, patients with low MYH9 expression levels in their ccRCC had a more significant improvement in PFS after receiving sunitinib when compared with those in patients with high MYH9 expression (Fig. 8e). Thus, MYH9 expression of tumor could serve as a valuable predictor of the prognosis for sunitinib-treated ccRCC patients.

## Discussion

Patients with indolent and aggressive ccRCC got a quite different prognosis, so identifying novel biomarkers for ccRCC could be of great significance. In this study, we used scRNA-seq to demonstrate that MYH9 was upregulated in the ccRCC cell subgroup. Additionally, high MYH9 expression predicted a poor prognosis of ccRCC patients. Subsequently, functional experiments showed that MYH9 promoted proliferation, migration of ccRCC cells in vitro and in vivo, which suggested that MYH9 might act as a cancer promoter in ccRCC. Moreover, MYH9 promoted ccRCC sunitinib resistance in vitro and MYH9 expression was closely related with the sunitinib response of ccRCC patients. Our study also showed that the role of MYH9 in ccRCC was partially associated with its ability to activate AKT signaling pathway, since it was well documented that the effects of inhibiting ccRCC of shMYH9 could be reversed by AKT agonist. As far as we know, no empirical research has ever been reported as to whether MYH9 affects the progression and sunitinib resistance of ccRCC via AKT.

Tumor heterogeneity, such as histological, cellular, and molecular/genetic heterogeneity, poses a major challenge to both accurate diagnosis and treatment of cancer, so it has recently become a hotbed of cancer research [17]. ccRCC is a typical example of heterogeneous cancer, which is featured with diverse clinical behavior [8]. scRNA-seq provides high-throughput and high-resolution transcriptomic analyses of individual cells. It allows heterogeneous cells to be sequenced individually to reveal the unique and subtle changes of the cell subtypes [18]. To investigate the underlying biology, we conducted a scRNA-seq of cells from two primary ccRCC. Here, 5 cell clusters in ccRCC tissues and adjacent normal tissues were identified by scRNA-seq clustering analysis. In order to investigate the development of ccRCC, we analyzed the DEGs in clusters 1 (renal tube cell) and 3 (cancer cell). Then, we identified 8 DEGs (TPM2,CPE,MYH9,A2M,FN1,SERPING1, COL6A1 and PGF). The expression level of these DEGs in cancer cell was significantly higher than that in renal tube cell, which was similar to our observations based on the databases. Combining the results of scRNA-seq and multiple database analyses, we ultimately chose MYH9 to further explore its role in ccRCC development. MYH9 takes part in many cellular movements, including cell adhesion, migration and division. Due to its actin-cross-linking and contractile function, MYH9 plays an important role in the maintenance of cytoskeletal structure and muscle tension [19]. Since cellular movements are closely associated with cancer progression and metastasis, the relationship between MYH9 and cancers has been investigated. Previous studies have reported that MYH9 exerted paradoxical roles in different cancers [20]. MYH9 acted as a suppressor gene in thyroid cancer by binding to the promoter region of lncRNA PTCSC2 and promoting forehead box E1 activation [11]. Additionally, Schramek et al reported that MYH9 could also suppress the progression of squamous cell carcinomas by regulating post-transcriptional p53 stabilization [15]. However, there were still many other investigations reported that MYH9 played the totally opposite role in other cancers. Betapudiet al found that depletion of MYH9 could inhibit MLCK and Rho Kinase, as well as the depression of migration capacity of breast cancer cells [21]. Another study reported MYH9 significantly enhanced MAPK/AKT pathway, and was remarkably associated with poor prognosis in colorectal cancer [13]. MYH9 was also regarded as an oncogene in esophageal squamous cancer, non-small cell lung cancer, and gastric cancer [22–25]. However, the role of MYH9 in ccRCC remains unknown. In our study, we found MYH9 was closely related to ccRCC proliferation and migration in vitro and in vivo. By investigating ccRCC patients’ cohort, we found that MYH9 was overexpressed in ccRCC samples compared with adjacent normal tissues and MYH9 was correlated with poor prognosis of ccRCC patients. In vitro, cell proliferation and migration study using shRNA showed weakened proliferation and migration abilities, while the study using LV-MYH9 transfection revealed enhanced the above abilities. In vivo, we tested the function of MYH9 in a xenograft tumor model and our study found transfection of LV-MYH9 into 786-O cells led to significantly increased volume and weight of xenograft tumors. Therefore, we might come to the conclusion that MYH9 acted as an important tumor promoter in ccRCC by promoting tumor proliferation and migration, but the molecular mechanism was not fully elucidated.

We thus sought to investigate the underlying mechanisms of MYH9-mediated cellular proliferation and migration of ccRCC. Firstly, we used KEGG and WIKI pathway analysis to show MYH9 enrichment in PI3K-AKT signaling. MYH9 expression showed a strong positive relationship with AKT1 expression in RCC tissues. In the current study, we found AKT signaling pathway was activated when MYH9 was overexpressed in vivo and in vitro. In fact, AKT acts as a key factor in regulating cellular survival, proliferation, migration, and angiogenesis [26]. Overexpression or activation of AKT has been reported in a variety of cancers, such as RCC, breast, prostate, and gastric cancers [27, 28]. Then, we explored whether the effects of MYH9 on ccRCC were mediated by AKT, and found this was the case. Pretreatment with the AKT agonist SC79 reversed the reduced capacities of proliferation and migration in MYH9 knockdown ccRCC cells. These results indicated that MYH9 promoted the proliferation and migration, at least in part via AKT signaling pathway in ccRCC. Our finding might provide not only a new biomarker, but also a potential target for ccRCC.

Sunitinib is the first-line therapy for advanced ccRCC, but its clinical application and curative effect have been limited by frequent drug resistance [29, 30]. Preliminary evidence showed that activation of alternative RTKs may contribute largely to therapeutic resistance and accumulating reports determined an increase of AKT activity in sunitinib-refractory ccRCC [31]. Interestingly, we found that up-regulated MYH9 could facilitate sunitinib-resistance of ccRCC cells and predict poor response to sunitinib for ccRCC patients. These data suggested that MYH9 levels might be of a valuable predictor of the response to sunitinib in ccRCC patients.

In conclusion, our data revealed that MYH9 was a key contributor for ccRCC carcinogenesis and sunitinib resistance. We provided evidence that MYH9 might activate AKT signaling pathway, further promoting ccRCC progression and sunitinib resistance. Our study contributed to a better understanding of ccRCC progression and provided a novel therapeutic target and prognostic predictor for ccRCC.

## Materials and methods

### Acquisition of cell samples and ccRCC population cohorts

We obtained the raw data of 23150 cell samples with single-cell transcriptome profiling from GSE152938 via the Gene Expression Omnibus (GEO) database (https://www.ncbi.nlm.nih.gov/geo/). The ccRCC tumor cells and adjacent tissue cells were finally analyzed in our study after filtering out poor-quality cells. We then merged the transcriptome data into one matrix and conducted the normalization process using the Matrix package. Normalization and additional analysis were performed by Seurat R package

### Processing of single-cell RNA-seq data

We utilized the Seurat package to generate the object and filtered out cells with poor quality. The reading depth of scRNA-seq was 10x genomics based on Illumina HiSeq 2500. Then, we conducted standard data preprocessing, where we calculated the percentage of the gene numbers, cell counts and mitochondria sequencing count. We excluded genes with less than only 10 cells detected and disregarded cells with less than 200 detected gene numbers. The proportion of mitochondria was restricted to less than 5%. Afterwards, we identified the gene symbols with significant differences across cells and constructed a characteristic variance diagram. In addition, we conducted PCA with linear dimensionality reduction and identified the significantly available dimensions of data sets with an estimated P value. Importantly, we further utilized the t-SNE algorithm to conduct the cluster classification analysis across cell samples and screened the marker genes between clusters with logFC = 0.5 and adjPval = 0.05 as the cutoff criteria. The heatmap of the top significant marker genes was illustrated via ggsci package. Finally, we used the marker genes to annotate the cluster and cell categories based on the SingleR package.

### Patient population

In all, ccRCC patients who underwent surgical resection in Qianfoshan Hospital, Shandong, China from 2014 to 2019 were included in this study. One cohort included 19 ccRCC tissues and paired adjacent kidney tissues. The second cohort included 42 ccRCC tissues and paired adjacent kidney tissues. The third cohort included 98 ccRCC tissues. Another independent cohort included 50 sunitinib-treated and 50 nonsunitinib-treated paired ccRCC tissues.

In that, patient inclusion criteria of sunitinib treated (*n* = 50) and nonsunitinib treated paired (*n* = 50) ccRCC tissues comprised a diagnosis of ccRCC with no prior anti-VEGF therapy. Patients received treatment with sunitinib, or not. Patients who received prior immunotherapy (that is, received their targeted therapy as second-line treatment) also were included. Patients treated initially with temsirolimus (an inhibitor of mammalian target of rapamycin), an investigational agent (that is, PTK787, AZD2171, pazopanib), or an investigational combination (for example, bevacizumab plus erlotinib, bevacizumab plus sorafenib) were excluded. Presence of nodal and metastatic disease was defined according to intraoperative, pathologic and radiographic findings. Patients were staged using radiographic reports and postoperative pathological data and were reassigned according to the 2017 AJCC TNM classification. Response to sunitinib of ccRCC patients were determined by computed tomography or magnetic resonance imaging, clinical progression, or death, with the use of the Response Evaluation Criteria in Solid Tumors (RECIST) version 1.1.

### Statistical analysis

All statistical analyses in this study were performed using SPSS 17.0 software (IBM, Armonk, NY, USA). Data were expressed as the means ± SD. The variance is similar between the groups that are being statistically compared. The significance of differences between the mean values of two groups were analysed by Student’s t-test. Linear correlations were performed by Pearson correlation analysis. The relation between the clinical variables was evaluated by Chi-square test. Kaplan-Meier method was utilized to compare the survival rate of ccRCC based on dichotomized MYH9 expression by a log-rank test. Blots presented are representation of at least three experiments. Statistical significance was indicated by p values less than 0.05. *p < 0.05, **p < 0.01, and ***p < 0.001.

## Supplementary information

supplementary materials

ARRIVE Guidelines Checklist

Ethical approval form

## Data Availability

The datasets generated for this study can be found in the GEO database (https://www.ncbi.nlm.nih.gov/geo/). All the data generated or analyzed during this study are included in this article and its supplementary information files or available from the author upon reasonable request.

## References

[1] Siegel RL, Miller KD, Jemal A (2020). Cancer statistics, 2020. CA: A Cancer J Clinicians.

[2] Capitanio U, Bensalah K, Bex A, Boorjian SA, Bray F, Coleman J (2019). Epidemiology of renal cell carcinoma. Eur. Urol.

[3] Capitanio U, Montorsi F (2016). Renal cancer. Lancet.

[4] Unverzagt S, Moldenhauer I, Nothacker M, Roßmeißl D, Hadjinicolaou AV, Peinemann F (2017). Immunotherapy for metastatic renal cell carcinoma. Cochrane Database Syst. Rev.

[5] Huang H, Gao Y, Liu A, Yang X, Huang F, Xu L (2019). EIF3D promotes sunitinib resistance of renal cell carcinoma by interacting with GRP78 and inhibiting its degradation. EBioMedicine.

[6] Luecken MD, Theis FJ (2019). Current best practices in single-cell RNA-seq analysis: a tutorial. Mol Syst Biol.

[7] Young MD, Mitchell TJ, Vieira Braga FA, Tran MGB, Stewart BJ, Ferdinand JR (2018). Single-cell transcriptomes from human kidneys reveal the cellular identity of renal tumors. Science.

[8] Zhang C, He H, Hu X, Liu A, Huang D, Xu Y (2019). Development and validation of a metastasis-associated prognostic signature based on single-cell RNA-seq in clear cell renal cell carcinoma. Aging.

[9] Pecci A, Ma X, Savoia A, Adelstein RS (2018). MYH9: Structure, functions and role of non-muscle myosin IIA in human disease. Gene.

[10] Cechova S, Dong F, Chan F, Kelley MJ, Ruiz P, Le TH (2018). MYH9 E1841K mutation augments proteinuria and podocyte injury and migration. J Am Soc Nephrology: JASN.

[11] Wang Y, He H, Li W, Phay J, Shen RL, Yu LB (2017). MYH9 binds to lncRNA gene PTCSC2 and regulates FOXE1 in the 9q22 thyroid cancer risk locus. Proc Natl Acad Sci USA.

[12] Ye G, Huang K, Yu J, Zhao L, Zhu X, Yang Q (2017). MicroRNA-647 Targets SRF-MYH9 axis to suppress invasion and metastasis of gastric cancer. Theranostics.

[13] Wang B, Qi X, Liu J, Zhou R, Lin C, Shangguan JJ (2019). MYH9 promotes growth and metastasis via activation of MAPK/AKT signaling in colorectal cancer. J Cancer.

[14] Surcel A, Schiffhauer ES, Thomas DG, Zhu QF, DiNapoli KT, Herbig M (2019). Targeting mechanoresponsive proteins in pancreatic cancer: 4-Hydroxyacetophenone blocks dissemination and invasion by activating MYH14. Cancer Res.

[15] Schramek D, Sendoel A, Segal JP, Slobodan Beronja S, Evan Heller E, Oristian D (2014). Direct in vivo RNAi screen unveils myosin IIa as a tumor suppressor of squamous cell carcinomas. Science.

[16] Chandarlapaty S (2012). Negative feedback and adaptive resistance to the targeted therapy of cancer. Cancer Discov..

[17] Khatib S, Pomyen Y, Dang H, Wang XW (2020). Understanding the cause and consequence of tumor heterogeneity. Trends Cancer.

[18] Maynard A, McCoach CE, Rotow JK, Harris L, Haderk F, Kerr DL (2020). Therapy-induced evolution of human lung cancer revealed by single-cell RNA sequencing. Cell.

[19] Kang JS, Lee SJ, Lee JH, Kim JH, Son SS, Cha SK (2019). Angiotensin II-mediated MYH9 downregulation causes structural and functional podocyte injury in diabetic kidney disease. Sci Rep.

[20] Wang Y, Liu S, Zhang Y, Yang J (2019). Myosin heavy chain 9: Oncogene or tumor suppressor gene?. Med Sci Monit: Int Med J Exp Clin Res.

[21] Betapudi V, Licate LS, Egelhoff TT (2006). Distinct roles of nonmuscle myosin II isoforms in the regulation of MDA-MB-231 breast cancer cell spreading and migration. Cancer Res.

[22] Katono K, Sato Y, Jiang SX, Kobayashi M, Nagashio R, Ryuge S (2015). Prognostic significance of MYH9 expression in resected non-small cell lung cancer. PloS One.

[23] Liu D, Zhang L, Shen Z, Tan F, Hu YF, Yu J (2012). Clinicopathological significance of NMIIA overexpression in human gastric cancer. Int J Mol Sci.

[24] Xia ZK, Yuan YC, Yin N, Yin BL, Tan ZP, Hu YR (2012). Nonmuscle myosin IIA is associated with poor prognosis of esophageal squamous cancer. Diseases of the esophagus: official journal of the International Society for. Dis Esophagus.

[25] Xiong D, Ye YL, Chen MK, Qin ZK, Li MZ, Zhang H (2012). Non-muscle myosin II is an independent predictor of overall survival for cystectomy candidates with early-stage bladder cancer. Oncol Rep.

[26] Song M, Bode AM, Dong Z, Lee MH (2019). AKT as a therapeutic target for cancer. Cancer Res.

[27] Hoxhaj G, Manning BD (2020). The PI3K-AKT network at the interface of oncogenic signalling and cancer metabolism. Nat Rev Cancer.

[28] Revathidevi S, Munirajan AK (2019). Akt in cancer: Mediator and more. Semin Cancer Biol.

[29] Adelaiye-Ogala R, Budka J, Damayanti NP, Arrington J, Ferris M, Hsu CC (2017). EZH2 modifies sunitinib resistance in renal cell carcinoma by kinome reprogramming. Cancer Res.

[30] Sun M, Marconi L, Eisen T, Escudier B, Giles RH, Haas NB (2018). Adjuvant vascular endothelial growth factor-targeted therapy in renal cell carcinoma: A systematic review and pooled analysis. Eur Urol.

[31] Han F, Li CF, Cai Z, Cai Z, Zhang X, Jin GX (2018). The critical role of AMPK in driving Akt activation under stress, tumorigenesis and drug resistance. Nat Commun..

