# Supplementary material for: Single-cell RNA-sequencing analysis reveals MYH9 promotes renal cell carcinoma development and sunitinib resistance via AKT signaling pathway
Source: Cell Death Discov. 2022 Mar 22;8:125. doi: 10.1038/s41420-022-00933-6 (PMC8941107; doi:10.1038/s41420-022-00933-6)
Supplement: Supplementary file 1 — supplementary materials [file 41420_2022_933_MOESM1_ESM.pdf]

Materials and methods

Oncomine analysis

The mRNA expression and DNA copy number of MYH9 in RCC were analyzed within the Oncomine 4.5 database. This analysis drew on a series of RCC studies, including Jones Renal (GSE15641), Beroukhim Renal (GSE14994), TCGA Renal, Lenburg Renal (GSE781) and Gumz Renal studies (GSE6344) (17-20). MYH9 expression was assessed in RCC tissue relative to its ex-pression in normal tissue, and differences associated with $p < 0.05$ were considered significant.

Lentiviral vector and infection

Lentiviral vector and infection were performed as previously described.(Qu, et al., 2016) The lentivirus encoding EGFP, EGFP-MYH9 plasmids were packaged and purified at HanBio Biotechnology (Shanghai, China) and infected cells following the manufacturer’s instructions.

Cell culture

ACHN and 786-O supplied by the Type Culture Collection of the Chinese Academy of Sciences (Shanghai, China) were cultured in Roswell Park Memorial Institute-1640. The cells were supplemented with 10% fetal bovine serum, 1% penicillin/streptomycin and cultured in humidified atmosphere of 5% CO_2 at 37 ℃ as previously described.(Xu, et al., 2017)

In vivo tumor xenograft experiment

A total of 10 male nude mice (BALB/c Nude; 4 weeks old) were purchased from the Vital River Laboratory Animal Technology Co. Ltd. (Beijing, China) and maintained

in a pathogen-free condition in accordance with relevant guidelines and regulations for the care and use of laboratory animals, with the approval of the Institutional Animal Care and Use Committee at Shandong Provincial Qianfoshan Hospital. 786-O cells (5×10^6 cells) in 100 μl PBS were implanted subcutaneously into the flanks of nude mice. Tumor size was monitored at 3 days intervals using calipers, and the tumor volumes were calculated according to the following formula: tumor volume = largest diameter $\times$ perpendicular height^2 $\times$ 0.5.

Western blotting analysis

Western blotting was performed as described previously(Xu, et al., 2016). Briefly speaking, RCC tissues and cells were homogenized in RIPA lysis buffer (Beyotime, China).Total protein was determined with a BCA protein assay kit (Pierce Biotechnology, Inc, Rockford, IL, USA). The lysates were analyzed by SDS-polyacrylamide gel electrophoresis and transferred electrophoretically to nitrocellulose membranes (Bio-Rad Corp, Hercules, CA). Blots were probed with antibodies specific for MYH9, p-AKT, AKT, PTEN, GAPDH. Antibodies for MYH9 (Cat. No. 3403), p-AKT (Cat. No. 4060), AKT (Cat. No. 9272), GAPDH (Cat. No. 5174) were obtained from Cell Signaling Technology (Boston, MA, USA). Immunoreactive proteins were revealed by an enhanced chemiluminescence (ECL) kit (Santa Cruz Biotechnology Inc, CA).

Wound healing assay

Cells were seeded into 6-well culture plates until they reached 90% confluency. Then, we used a sterile peptide tip to create wounds in the cell monolayer. Cell migration

was photographed using microscopy (magnification, ×50) at 0 h, 12 h, 24 h after injury.

Transwell migration assay

Cell transwell migration assays were performed with 24-well transwell chamber coated matrigel according to the manufacturer's instructions (Millipore, Billerica, MA, USA). Then, the migrated cells were counted by staining with 4'6-diamidino-2-phenylindole (DAPI) (magnification, ×200).

Plate colony formation assay

Cells were seeded into 6-well plates with a density of 500 cells/ml. The cells were incubated for 10 days at 37 ℃. Then, the cells were stained with 0.1% crystal violet for 15 min. Only colonies containing >50 cells were counted. Cells were photographed using microscopy to count the number of positive colonies.

Cell counting kit-8 (CCK8)

The cells were seeded in 96-well plates with a concentration of 5000 cells/well. 10μl CCK8 mixed with 100μl medium was added into each well. After 2 h of incubation at 37 ℃, the plates were read at a wave length of 450nm using microplate reader. All experiments were conducted in triplicate.

RNA extraction and real-time quantitative polymerase chain reaction(RT-PCR)

RT-PCR was performed as described previously(Xu, et al., 2017). Briefly, according to manufacturer's protocol, the total RNA was extracted from RCC tissue or cells with TRIzol reagent (Takara, Dalian, China). Reverse transcription reactions were performed using the PrimeScriptTM RT reagent Kit (Takara, Dalian, China). The

expression of genes were measured by PCR using SYBR® Premix Ex Taq (Takara, Dalian, China) and the PCR products were verified by ABI 7500 sequencer (Applied Biosystems, Foster City, CA, USA). The results for each sample were normalized to the values of β-actin.

The primer sequences were:

MYH9: 5'-AGTTTGTCTCGGAGCTGTGG-3'(forward)

5'-GGTTCGTGTTCCTCAGCGTA-3' (reverse),

β-actin: 5'-CTCTTCCAGCCTTCCTTCCT-3'(forward)

5'-AGCACTGTGTTGGCGTACAG-3' (reverse).

Dual luciferase reporter assay

AKT luciferase reporter plasmid (Promega, Madison, WI, USA) was transfected into cells using Lipofectgamine 3000 (Invitrogen, Carlsbad, CA, USA), and then was analyzed by Dual-Glo Luciferase Reporter Assays (Promega, Madison, WI, USA). After 48h of transfection, the relative luciferase activity was measured and quantified.

Immunohistochemistry (IHC) staining

Tumor sections were fixed with 10% buffered formalin, embedded in paraffin, sectioned at 5 μm thickness and used for IHC staining. Primary antibodies against MYH9 and p-AKT were used. After incubated for 1 h at 37 ℃, secondary staining kits were used and then hematoxylin restaining. The widely accepted German semi-quantitative scoring system was used to score staining intensity (no staining, 0; weak staining, 1; moderate staining, 2; and strong staining, 3) and extent (<5% = 0; 5–25% = 1; 26–50% = 2; 51–75% = 3; and 76–100% = 4) in the nucleus, cytoplasm,

and membrane. The final immunoreactivity score, which ranged from 0 (minimum) to 12 (maximum), was determined by multiplying the intensity and extent scores. The scores less than 6 were divided into low MYH9 expression group, while more than or equel to 6 were divided into high MYH9 expression group.

Supplementary figure legends

Supplementary Fig. 1 Single-cell quality control and dimension reduction cluster analysis. a, b Quality control of scRNA-seq for 3 samples. Cells with poor quality were filtered out and the percentage of mitochondrial genes were less than 5%. Positive associations between detected gene counts and sequencing depth were analyzed. **c** 3,000 hypervariable genes from all the genes shown in red and the top 10 hypervariable genes. **d, e, f** Principal component analysis (PCA) and cell groups were classified into five categories. **g** Each cell subgroup is presented in different samples.

Supplementary Fig. 2 Expression of MYH9, A2M, FN1, COL6A1, and PGF in various cell subgroups. a, b, and c The expression levels of MYH9, A2M, FN1, COL6A1, and PGF in each cell subgroup are shown with Feather plot, VlnPlot and RidgePlot.

Supplementary Fig. 3 Expression of MYH9, A2M, FN1, COL6A1, and PGF in ccRCC cells and normal kidney cells. The relative mRNA expression of MYH9, A2M, FN1, COL6A1, and PGF in ccRCC cells (786-O, A498, ACHN, and Caki-1) and normal kidney cells (HK2 and 293T) were tested by qRT-PCR.

Supplementary Fig. 4 Prognosis of RCC patients with different expression of MYH9. High MYH9 expression was associated with poor prognosis of patients with RCC.

Supplementary Fig. 5 Related to Figure 6. Genes differentially expressed in correlation with MYH9 in RCC (LinkedOmics). a A Pearson test was used to analyze correlations between MYH9 and genes differentially expressed in RCC. **b**

Heat maps showing genes positively and negatively correlated with MYH9 in RCC (TOP 50). Red indicates positively correlated genes and green indicates negatively correlated genes.

Supplementary Fig. 6 Related to Figure 6. Gene expression correlation analysis for MYH9, CSF1, AKT1, and CREB3L (LinkedOmics). The scatter plot shows Pearson correlation of RBM8A expression with expression of CSF1, AKT1, and CREB3L (LinkedOmics).

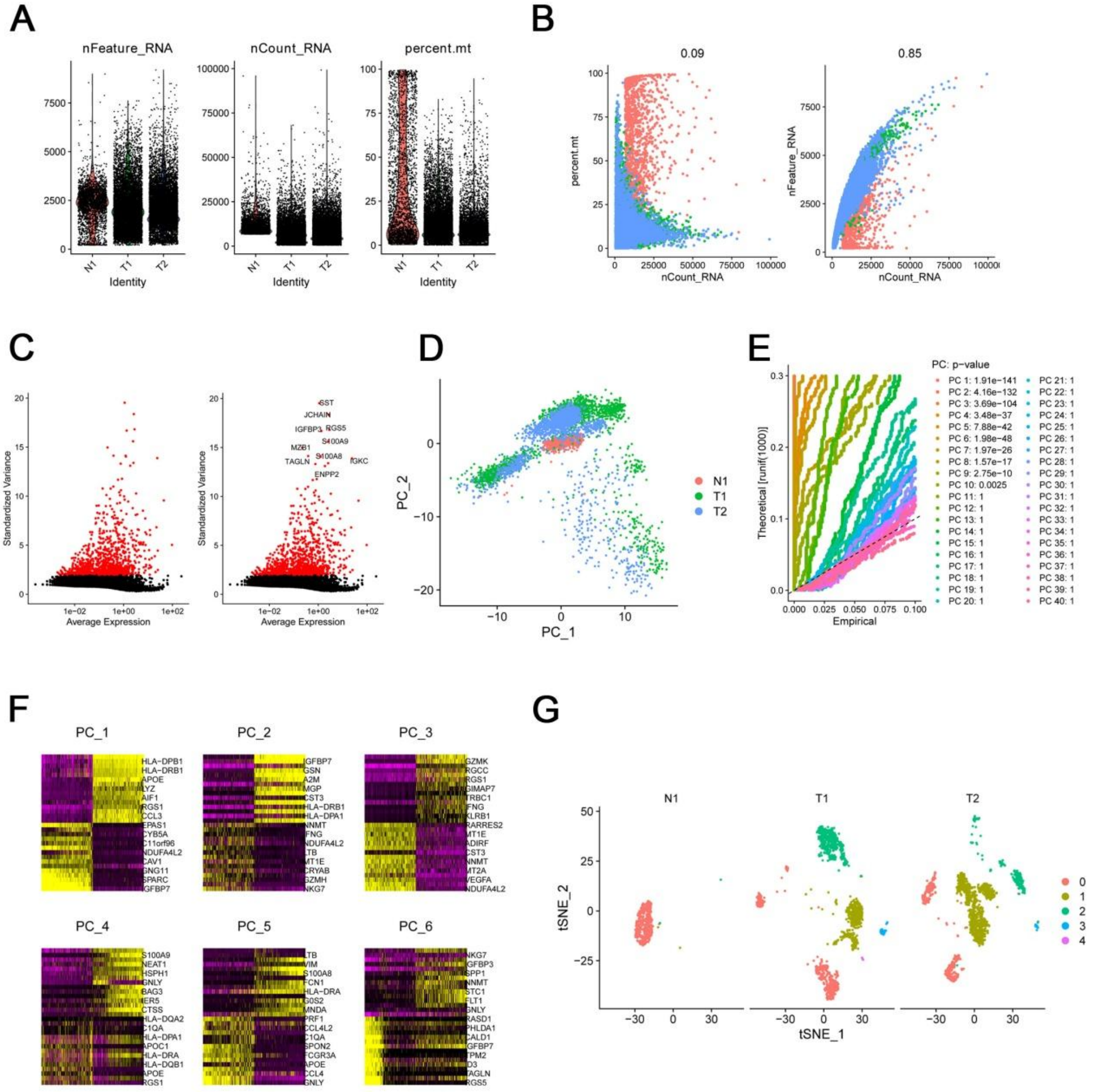

Supplementary Fig. 1 Single-cell quality control and dimension reduction cluster analysis.

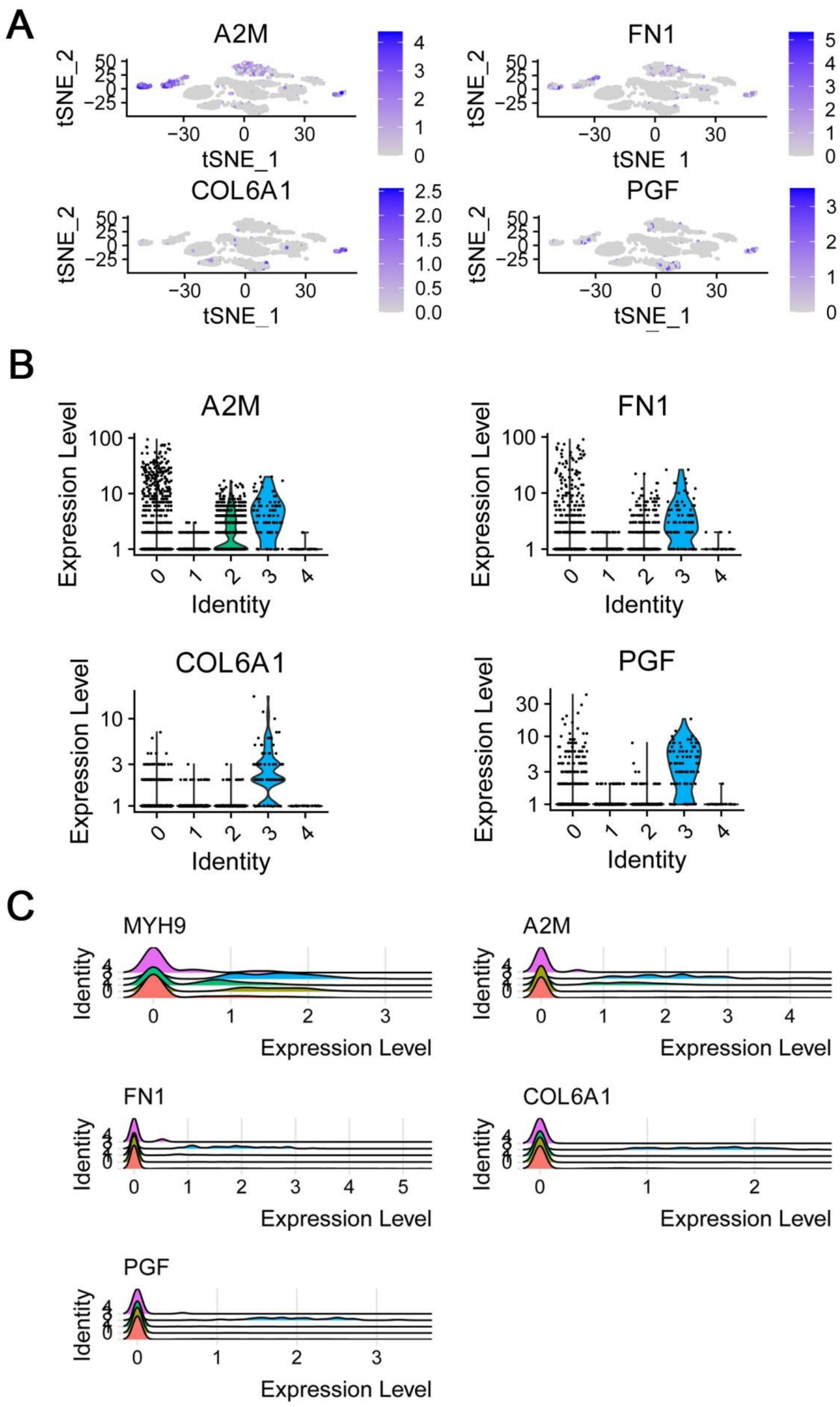

Supplementary Fig. 2 Expression of MYH9, A2M, FN1, COL6A1, and PGF in various cell subgroups.

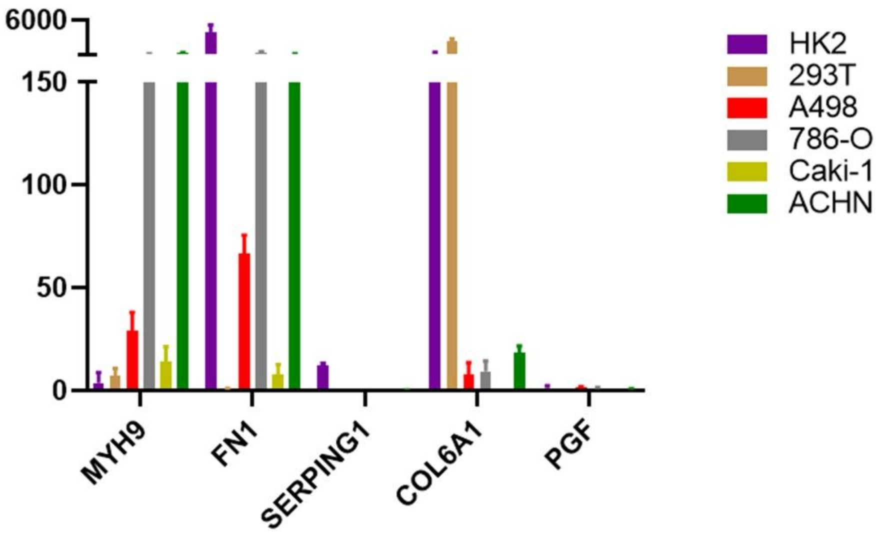

128

129 **Supplementary Fig. 3 Expression of MYH9, A2M, FN1, COL6A1, and PGF in**
130 **ccRCC cells and normal kidney cells.**

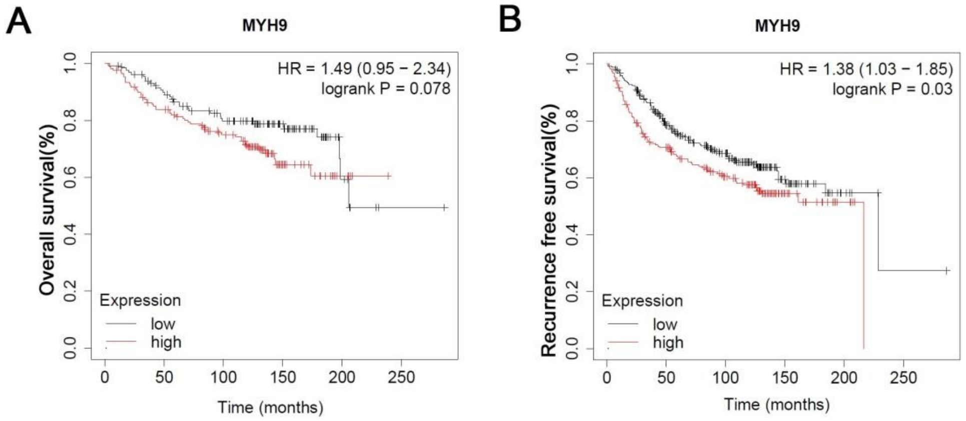

Supplementary Fig. 4 Prognosis of RCC patients with different expression of MYH9.

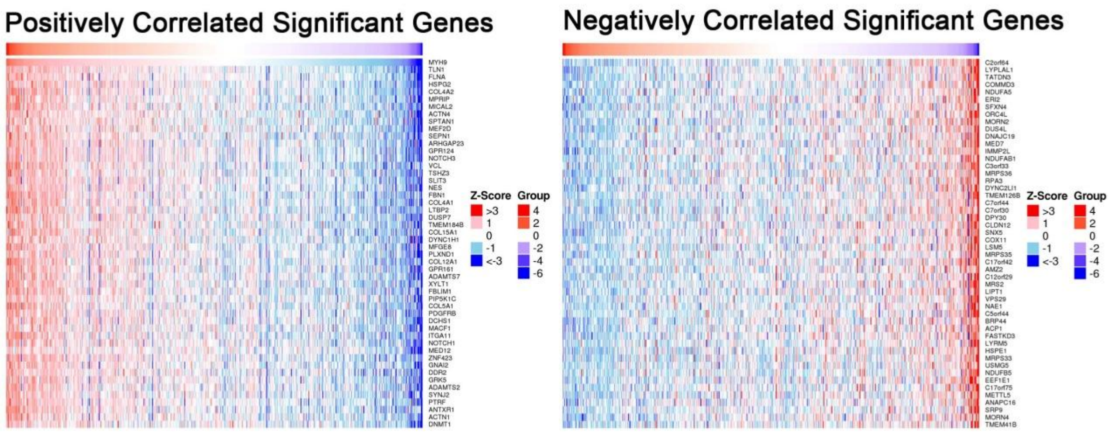

Supplementary Fig. 5 Related to Figure 6. Genes differentially expressed in correlation with MYH9 in RCC (LinkedOmics).

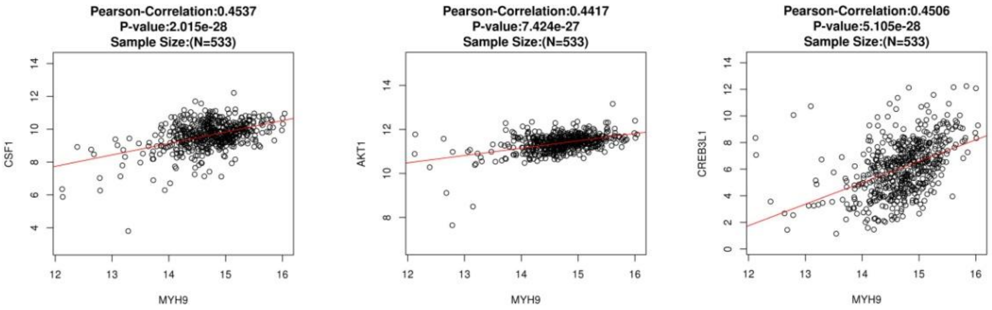

Supplementary Fig. 6 Related to Figure 6. Gene expression correlation analysis for MYH9, CSF1, AKT1, and CREB3L (LinkedOmics).

Supplemental Table 1. The DEGs between renal tube cell and renal cancer cell

Gene	pct.1	pct.2	avg_log2FC	p_val_adj
RGS5	0.085	1	-6.4573765	3.24E-146
TAGLN	0.022	0.855	-4.7899791	7.30E-203
TPM2	0.068	0.928	-3.8585574	1.22E-142
BGN	0.185	0.952	-3.642823	5.60E-81
RASD1	0.034	0.759	-3.4292992	4.67E-143
MYL9	0.171	0.952	-3.3386282	8.13E-85
PLAC9	0.028	0.952	-3.2734065	5.47E-218
MGP	0.224	0.988	-3.2514172	4.45E-66
FRZB	0.019	0.831	-3.1832397	2.24E-202
GJA4	0.061	0.88	-2.9975312	1.21E-132
PDGFRB	0.01	0.843	-2.8969567	1.58E-238
COL6A2	0.118	0.855	-2.7338797	3.09E-84
MAP1B	0.099	0.867	-2.618023	1.68E-96
LHFPL6	0.105	0.855	-2.6170049	1.19E-90
NOTCH3	0.011	0.855	-2.615018	3.75E-241
ITM2C	0.076	0.819	-2.4897488	2.40E-103
KCNE4	0.078	0.759	-2.4786136	3.93E-86
CPE	0.141	0.855	-2.4285144	7.62E-68
MFGE8	0.07	0.867	-2.4049221	3.87E-122
CRIP1	0.174	0.843	-2.4012799	1.51E-58
SOD3	0.068	0.819	-2.3658044	5.43E-113
MCAM	0.127	0.892	-2.3306642	3.06E-79
TINAGL1	0.161	0.831	-2.1986907	2.10E-61
COL18A1	0.28	0.867	-2.1738396	9.21E-44
PGF	0.109	0.795	-2.1592403	6.50E-70
ID4	0.273	0.771	-1.9570441	8.98E-30
COL4A2	0.211	0.88	-1.9500562	1.47E-45
LGALS3BP	0.229	0.783	-1.9321518	2.19E-38
COL6A1	0.101	0.795	-1.9139344	5.28E-80
TPM4	0.274	0.807	-1.7078411	3.29E-29
PMEPA1	0.222	0.759	-1.672586	2.49E-32
ARHGDIB	0.22	0.831	-1.6294165	9.05E-35
COL4A1	0.22	0.783	-1.4144894	3.05E-27
MYH9	0.254	0.759	-1.3079801	8.75E-25
SERPING1	0.273	0.759	-1.2053919	4.75E-21
FN1	0.14	0.783	-1.1062251	7.53E-46
A2M	0.225	0.855	-0.9173203	1.88E-26

Supplemental Table 2. Clinical characteristics of RCC patients cohort 1.

Patient ID	Age	Gender	Pathological diagnosis	Furman grade	TNM stage
1	64	F	ccRCC	IV	T1aN0M0
2	45	M	ccRCC	II	T1aN0M0
3	72	F	ccRCC	III	T3cN0M0
4	43	M	ccRCC	I-II	T1aN0M0
5	50	M	ccRCC	I	T1aN0M0
6	67	F	ccRCC	I	T1aN0M0
7	38	M	ccRCC	II	T1aN0M0
8	68	F	ccRCC	II	T1bN0M0
9	49	F	ccRCC	II	T1bN0M0
10	64	M	ccRCC	II	T1aN0M0
11	71	M	ccRCC	II	T1aN0M0
12	54	M	ccRCC	II	T3bN0M0
13	63	M	ccRCC	II	T1aN0M0
14	62	F	ccRCC	II	T1aN0M0
15	40	M	ccRCC	I-II	T1aN0M0
16	53	M	ccRCC	I	T1aN0M0
17	46	M	ccRCC	II	T1aN0M0
18	59	F	ccRCC	II-III	T3cN0M0
19	48	M	ccRCC	II	T1aN0M0
20	66	F	ccRCC	I	T1aN0M0
21	77	M	ccRCC	IV	T1aN0M0
22	62	M	ccRCC	II	T2aN0M0
23	76	M	ccRCC	II	T1aN0M0

24	68	F	ccRCC	II	T1aN0M0
25	30	F	ccRCC	III	T2aN0M0
26	60	M	ccRCC	II	T1aN0M0
27	54	M	ccRCC	II	T1aN0M0
28	48	M	ccRCC	II	T1aN0M0
29	78	M	ccRCC	III	T1aN0M1
30	64	M	ccRCC	II	T1aN0M0
31	60	F	ccRCC	I	T1aN0M0
32	82	M	ccRCC	II	T1bN0M1
33	58	F	ccRCC	IV	T2aN0M0
34	73	F	ccRCC	II	T1aN0M0
35	50	M	ccRCC	III	T1bN0M0
36	54	M	ccRCC	II	T2bN0M1
37	34	M	ccRCC	II	T1aN0M0
38	52	F	ccRCC	II-III	T1aN0M0
39	67	M	ccRCC	I	T1bN0M0
40	67	F	ccRCC	II	T1bN0M0
41	61	M	ccRCC	II	T1aN0M0
42	60	F	ccRCC	II	T1aN0M0

144

145 **Supplemental Table 3. The clinical characteristics of 98 RCC patients in**
146 **indicated groups (cohort 2)**

**Variables**	**All patients (n = 98)**		**p value**
	**Low MYH9 (n = 33)**	**High MYH9 (n = 65)**	
**Gender**			0.52
Male	22	39	
Female	11	26	
**Age**			0.43
≤60	23	39	
＞60	10	26	
**Furman grade**			0.32
Ⅰ/Ⅱ	26	35	
Ⅲ/Ⅳ	7	30	
**TNM stage**			0.53
Ⅰ/Ⅱ	29	54	
Ⅲ / Ⅳ	4	11	
**Distant metastasis**			0.99
no	32	63	
yes	1	2	

147

Supplemental Table 4. Clinical characteristics of RCC patients cohort 2.

Patient ID	Age	Gender	Pathological diagnosis	Furman grade	TNM stage
1	45	M	ccRCC	II	T1bN0M0
2	50	F	ccRCC	II	T1aN0M0
3	64	F	ccRCC	II	T1aN0M0
4	40	M	ccRCC	III	T1bN1M0
5	66	M	ccRCC	II	T1aN0M0
6	75	F	ccRCC	III	T3aN0M1
7	62	F	ccRCC	IV	T2aN0M0
8	46	M	ccRCC	II	T1bN0M0
9	57	F	ccRCC	III	T1bN0M0
10	71	F	ccRCC	IV	T3aN0M0
11	68	M	ccRCC	II	T3aN0M0
12	53	M	ccRCC	II	T1aN0M0
13	55	M	ccRCC	II	T1aN0M0
14	39	M	ccRCC	II	T1aN0M0
15	50	M	ccRCC	II	T1bN0M0
16	51	F	ccRCC	III	T1bN0M0
17	43	F	ccRCC	III	T4N1M0
18	41	M	ccRCC	III	T2aN0M0
19	46	M	ccRCC	II	T1bN0M0
20	46	M	ccRCC	III	T1aN0M0
21	45	M	ccRCC	II	T1bN0M0
22	37	F	ccRCC	I	T1bN0M0
23	73	F	ccRCC	II	T1aN0M0

24	71	M	ccRCC	III	T2aN0M0
25	55	M	ccRCC	II	T1bN0M0
26	41	M	ccRCC	II	T1bN0M0
27	63	F	ccRCC	I	T1bN0M0
28	53	F	ccRCC	II	T1bN0M0
29	75	F	ccRCC	II	T1bN0M0
30	46	F	ccRCC	II	T1aN0M0
31	81	M	ccRCC	II	T1bN1M0
32	42	F	ccRCC	I	T1bN0M0
33	45	M	ccRCC	I	T1aN0M0
34	76	M	ccRCC	II	T1bN0M0
35	61	F	ccRCC	II	T1bN0M0
36	44	M	ccRCC	II	T1bN0M0
37	53	M	ccRCC	III	T2aN1M1
38	41	M	ccRCC	I	T1aN0M0
39	73	M	ccRCC	III	T1bN0M0
40	46	F	ccRCC	IV	T4N0M0
41	40	M	ccRCC	I	T1aN0M0
42	46	M	ccRCC	II	T1bN0M0
43	61	M	ccRCC	II	T2bN0M0
44	25	M	ccRCC	II	T2bN0M0
45	57	F	ccRCC	III	T3bN0M0
46	21	F	ccRCC	II	T2bN0M0
47	54	F	ccRCC	III	T2aN0M0
48	64	M	ccRCC	III	T2aN0M0
49	63	F	ccRCC	III	T1bN0M0

50	68	M	ccRCC	II	T1aN0M0
51	53	M	ccRCC	I	T1bN0M0
52	60	M	ccRCC	III	T1bN0M0
53	69	M	ccRCC	III	T1bN0M0
54	61	F	ccRCC	III	T3N1M0
55	50	M	ccRCC	III	T4N0M1
56	64	F	ccRCC	II	T2bN0M0
57	68	M	ccRCC	III	T2bN0M0
58	47	M	ccRCC	III	T1bN0M0
59	42	F	ccRCC	II	T1aN0M0
60	63	F	ccRCC	II	T3N0M0
61	55	M	ccRCC	III	T1bN0M0
62	50	M	ccRCC	I	T1aN0M0
63	52	M	ccRCC	III	T3bN0M0
64	59	M	ccRCC	III	T1bN0M0
65	42	M	ccRCC	II	T2aN0M0
66	51	F	ccRCC	II	T2bN0M0
67	44	F	ccRCC	II	T1bN0M0
68	76	F	ccRCC	II	T2aN0M0
69	81	F	ccRCC	III	T1bN0M0
70	57	F	ccRCC	IV	T2aN0M0
71	43	M	ccRCC	II	T2bN0M0
72	53	M	ccRCC	III	T1bN0M0
73	74	M	ccRCC	I	T1bN0M0
74	61	M	ccRCC	II	T1bN0M0
75	67	M	ccRCC	III	T2aN0M0

76	71	M	ccRCC	II	T2aN0M0
77	42	M	ccRCC	II	T1aN0M0
78	41	M	ccRCC	III	T1aN0M0
79	52	F	ccRCC	II	T1aN0M0
80	36	M	ccRCC	III	T3bN0M0
81	64	M	ccRCC	I	T1aN0M0
82	58	M	ccRCC	II	T1aN0M0
83	58	M	ccRCC	II	T1aN0M0
84	51	M	ccRCC	II	T1bN0M0
85	57	M	ccRCC	I	T1aN0M0
86	77	F	ccRCC	I	T1aN0M0
87	55	M	ccRCC	III	T1bN0M0
88	58	M	ccRCC	I	T1bN0M0
89	49	M	ccRCC	II	T1bN0M0
90	48	M	ccRCC	III	T1bN0M0
91	37	F	ccRCC	II	T2aN0M0
92	61	F	ccRCC	II	T1aN0M0
93	72	F	ccRCC	IV	T4N1M0
94	57	F	ccRCC	II	T1bN0M0
95	67	F	ccRCC	II	T1bN0M0
96	60	M	ccRCC	III	T1aN0M0
97	61	M	ccRCC	II	T1aN0M0
98	72	M	ccRCC	III	T1aN0M0

149

Supplemental Table 5. Immunoreactivity score of the tissue microarray (cohort 2)

IMMUNOREACTIVITY SCORE (0-12)	ALL PATIENTS (N = 98)
0	2
1	7
2	4
3	9
4	11
6	35
8	26
12	4

Supplemental Table 6, related to Figure 8. The clinical characteristics of 100 ccRCCpatients (cohort 3) in indicated groups

Variables	Low MYH9(n = 50)		pvalue	High MYH9 (n = 50)		p value	All patients(n = 100)		p value
	Control (n = 25)	Sunitinib(n = 25)		Control (n = 25)	Sunitinib(n = 25)		Control (n = 50)	Sunitinib(n = 50)	
**Gender**			0.771			0.713			1.000
Male	16	15		20	21		36	36	
Female	9	10		5	4		14	14	
**Age**			0.47			0.765			0.829
≤60	19	18		19	17		35	34	
＞60	6	7		6	8		15	16	
**Furman grade**			0.771			0.254			0.316
Ⅰ/Ⅱ	10	9		12	9		26	21	
Ⅲ/Ⅳ	15	16		13	16		24	29	
**TNM stage**			0.556			0.564			0.410
Ⅰ/Ⅱ	8	10		9	11		17	21	
Ⅲ / Ⅳ	17	15		16	14		33	29	
**Distant metastasis**			0.390			0.564			0.317
no	13	16		9	11		22	27	
yes	12	9		16	14		28	23	

Supplemental Table 7. Clinical characteristics of RCC patients (cohort 3)

Patient ID	Age	Gender	Pathological diagnosis	Furman grade	TNM stage	Sunitinib	Response to sunitinib
1	77	M	ccRCC	IV	T2aN0M1	Yes	PD
2	51	M	ccRCC	IV	T2aN0M1	Yes	PD
3	48	M	ccRCC	IV	T3aN1M1	Yes	PD
4	29	F	ccRCC	IV	T2aN0M1	Yes	PD
5	25	F	ccRCC	IV	T4N1M1	Yes	SD
6	63	M	ccRCC	III	T2aN1M1	Yes	PD
7	59	F	ccRCC	III	T2aN0M1	Yes	PR
8	51	M	ccRCC	III	T1aN0M1	Yes	PD
9	61	M	ccRCC	II	T2bN0M1	Yes	SD
10	63	F	ccRCC	II	T2aN0M1	Yes	PR
11	58	M	ccRCC	II	T1bN1M1	Yes	PD
12	64	M	ccRCC	IV	T3aN0M1	Yes	PR
13	52	M	ccRCC	II	T3aN0M1	Yes	PD

14	51	M	ccRCC	II	T3aN0M1	Yes	PD
15	72	M	ccRCC	III	T2aN0M0	Yes	PR
16	65	M	ccRCC	III	T2bN0M0	Yes	PD
17	48	M	ccRCC	III	T2aN0M0	Yes	PD
18	45	M	ccRCC	III	T2bN0M0	Yes	PR
19	75	M	ccRCC	II	T2aN0M0	Yes	PD
20	42	M	ccRCC	I	T2aN0M0	Yes	PD
21	53	M	ccRCC	IV	T1bN0M0	Yes	PR
22	46	M	ccRCC	IV	T1bN0M0	Yes	SD
23	58	M	ccRCC	III	T1aN0M0	Yes	PR
24	63	M	ccRCC	II	T1bN0M0	Yes	PR
25	46	M	ccRCC	I	T1aN0M0	Yes	PD
26	61	F	ccRCC	IV	T1aN0M1	Yes	SD
27	52	M	ccRCC	IV	T2aN0M1	Yes	SD
28	50	M	ccRCC	II	T2aN0M1	Yes	PD
29	43	M	ccRCC	IV	T2aN0M1	Yes	PR

30	36	M	ccRCC	IV	T2aN0M1	Yes	PR
31	54	M	ccRCC	III	T1bN0M1	Yes	PD
32	73	F	ccRCC	II	T4N0M1	Yes	PD
33	64	F	ccRCC	II	T1bN0M1	Yes	PR
34	50	F	ccRCC	II	T2aN0M1	Yes	PD
35	70	M	ccRCC	IV	T2aN1M0	Yes	PD
36	51	M	ccRCC	IV	T3aN0M0	Yes	PR
37	49	F	ccRCC	III	T3aN0M0	Yes	PR
38	70	M	ccRCC	II	T3aN0M0	Yes	PD
39	59	M	ccRCC	II	T3aN0M0	Yes	PD
40	58	F	ccRCC	II	T3bN0M0	Yes	PR
41	77	M	ccRCC	III	T2aN0M0	Yes	PR
42	52	F	ccRCC	II	T2aN0M0	Yes	PR
43	44	M	ccRCC	III	T2aN0M0	Yes	PD
44	43	F	ccRCC	III	T2aN0M0	Yes	PR
45	37	F	ccRCC	IV	T1aN0M0	Yes	PR

46	73	M	ccRCC	III	T1bN0M0	Yes	PR
47	54	F	ccRCC	II	T1aN0M0	Yes	PD
48	57	M	ccRCC	II	T1aN0M0	Yes	PR
49	43	M	ccRCC	I	T1bN0M0	Yes	PR
50	42	M	ccRCC	I	T1aN0M0	Yes	PR
51	64	M	ccRCC	IV	T2aN0M1	No	
52	59	M	ccRCC	IV	T3aN0M1	No	
53	57	M	ccRCC	IV	T3aN0M1	No	
54	56	M	ccRCC	IV	T2aN0M1	No	
55	50	F	ccRCC	IV	T4N1M1	No	
56	49	M	ccRCC	II	T1aN0M1	No	
57	40	F	ccRCC	IV	T3aN0M1	No	
58	67	F	ccRCC	IV	T3bN0M1	No	
59	60	M	ccRCC	III	T4N0M1	No	
60	55	M	ccRCC	II	T2aN0M1	No	
61	61	F	ccRCC	II	T4N1M1	No	

62	51	M	ccRCC	IV	T3aN0M1	No
63	59	M	ccRCC	III	T3aN1M1	No
64	44	M	ccRCC	III	T3aN0M1	No
65	71	M	ccRCC	II	T2aN1M1	No
66	54	F	ccRCC	II	T3aN0M1	No
67	74	M	ccRCC	II	T2aN0M0	No
68	69	M	ccRCC	II	T2aN0M0	No
69	52	M	ccRCC	II	T2bN0M0	No
70	49	M	ccRCC	II	T2aN0M0	No
71	42	M	ccRCC	II	T2aN0M0	No
72	53	M	ccRCC	II	T1bN0M0	No
73	70	M	ccRCC	I	T1aN0M0	No
74	57	M	ccRCC	I	T1bN0M0	No
75	48	M	ccRCC	I	T1bN0M0	No
76	62	F	ccRCC	IV	T1aN1M1	No
77	50	M	ccRCC	IV	T2bN0M1	No

78	49	F	ccRCC	IV	T2aN0M1	No
79	43	F	ccRCC	IV	T1bN1M1	No
80	75	M	ccRCC	III	T2aN1M1	No
81	68	F	ccRCC	II	T3bN0M1	No
82	52	F	ccRCC	III	T1aN1M1	No
83	52	M	ccRCC	III	T2bN0M1	No
84	45	F	ccRCC	III	T1aN0M1	No
85	41	M	ccRCC	III	T2bN0M1	No
86	55	M	ccRCC	II	T2aN0M1	No
87	72	F	ccRCC	IV	T3aN0M0	No
88	68	M	ccRCC	IV	T3bN0M1	No
89	36	M	ccRCC	IV	T3bN0M0	No
90	69	M	ccRCC	I	T2aN1M0	No
91	59	F	ccRCC	III	T1aN1M0	No
92	51	M	ccRCC	II	T3aN0M0	No
93	50	M	ccRCC	II	T2aN0M0	No

94	57	M	ccRCC	I	T2aN0M0	No
95	66	M	ccRCC	II	T1aN0M0	No
96	69	M	ccRCC	II	T1aN0M0	No
97	55	M	ccRCC	I	T1aN0M0	No
98	54	M	ccRCC	I	T1aN0M0	No
99	46	F	ccRCC	I	T1aN0M0	No
100	41	M	ccRCC	I	T1bN0M0	No
