# Supplementary material for: Single-cell RNA-sequencing analysis reveals MYH9 promotes renal cell carcinoma development and sunitinib resistance via AKT signaling pathway
Source: Cell Death Discov. 2022 Mar 22;8:125. doi: 10.1038/s41420-022-00933-6 (PMC8941107; doi:10.1038/s41420-022-00933-6)
Supplement: Supplementary file 3 — Ethical approval form [file 41420_2022_933_MOESM3_ESM.pdf]

地址：济南市经十路 16766 号　　邮编：250014　　电话：0531-89268217　传真：0531-82963647

山东大学附属千佛山医院医学伦理委员会审批报告

【2020】伦审字（S399）号

项目名称	Myosin heavy chain 9 promotes renal cell carcinoma development and sunitinib resistance via AKT signaling pathway		
申请单位	山东大学附属千佛山医院		
项目负责人	王建宁	职称	主任医师
报送资料	课题研究方案	有■	无□
	知情同意书	有■	无□
	观察记录表	有■	无□
	研究人员名单	有■	无□
审查	研究者资格	符合要求■	不符合要求□
	课题研究方案	适当■	不适当□
	获取知情同意书方法	适当■	不适用□

审评意见：

本伦理委员会审阅并讨论了上述相关资料，该课题研究符合《世界医学大会赫尔辛基宣言》，经伦理委员会审核，同意该课题实施。

山东大学附属千佛山医院医学伦理委员会

日期：2020 年 11 月 29 日
